# The role of T-helper and T regulatory cells in driving neutrophilic and eosinophilic inflammation in bronchiectasis

**DOI:** 10.3389/fimmu.2025.1598257

**Published:** 2025-06-16

**Authors:** Evangelia Fouka, Anders Lindén, Apostolos Bossios

**Affiliations:** ^1^ Division for Lung and Airway Research, Institute of Environmental Medicine, Karolinska Institutet, Stockholm, Sweden; ^2^ Lung Laboratory, Center for Molecular Medicine, Karolinska University Hospital, Stockholm, Sweden; ^3^ National and Kapodistrian University of Athens, 2nd Department of Respiratory Medicine, University General Hospital Attikon, Athens, Greece; ^4^ Karolinska Severe COPD Center, Department of Respiratory Medicine and Allergy, Karolinska University Hospital, Stockholm, Sweden; ^5^ Karolinska Severe Asthma Center, Department of Respiratory Medicine and Allergy, Karolinska University Hospital, Stockholm, Sweden

**Keywords:** bronchiectasis, neutrophilic inflammation, eosinophilic inflammation, T helper cells, T regulatory cells, microbiome, endotypes

## Abstract

Bronchiectasis is a chronic airway disease characterized by dysbiosis, persistent inflammation, and permanent structural airway damage. Neutrophilic inflammation is a key pathogenic feature, as indicated by enhanced neutrophil-derived proteases and formation of neutrophil extracellular traps (NETs), associated with poor prognosis. However, recent studies have identified an eosinophilic endotype in up to 30% of patients, characterized by higher levels of type 2 (T2) cytokines and fractional exhaled nitric oxide (FeNO). The role of T helper (Th) cells in the dysregulated inflammatory environment of bronchiectasis remains unclear. Evidence suggests that persistent bacterial infection can skew adaptive immunity from Th1 toward Th2 response, while the airway microbiome-IL-17 axis is also a critical regulator of chronic inflammation. T regulatory (Treg) cells have been shown to play a protective role against excessive chronic inflammation by modulating the function of several types of effector cells, including the Th17 subset. However, the capacity of this subset to delay or prevent disease progression remains to be determined Microbial dysbiosis, with loss of diversity and increased quantity of bacterial pathogens, may also be important for disease progression, and emerging evidence indicates that distinct inflammatory endotypes associate with specific microbiota alterations, especially in severe disease. In this review, we provide an overview of the immune cells and cytokine signaling that are involved in the pathogenesis of bronchiectasis. Additionally, we present the main endotypes of bronchiectasis and explore the relationships between the type of inflammation and alterations in microbiota, as well as the potential benefits of targeting specific pathophysiological mechanisms for the management of bronchiectasis. This review also examines how bacterial infection can shift adaptive immunity from Th1 toward Th2 responses, the role of the airway microbiome-IL-17 axis in chronic inflammation and the potential protective role of Treg cells against excessive inflammation. Novel therapeutic strategies are highlighted, with focus on targeting specific cytokine signaling pathways and restoring Th17/Treg balance These developments underscore a shift toward precision medicine in bronchiectasis, emphasizing the importance of identifying specific inflammatory endotypes to tailor treatment strategies effectively.

## 1 Introduction

Bronchiectasis is a chronic airway disease characterized by permanent and abnormal dilation of the bronchi and clinically by persistent cough and sputum production that periodically worsen due to acute respiratory infections, known as “exacerbations” (1). Typical structural airway changes include thickening of the bronchial walls, loss of normal ciliary function, mucus plugging, and the formation of sac-like or cylindrical bronchial dilations (2). Although the exact prevalence of bronchiectasis remains uncertain, a report in 2013 indicated an increase of 40% since 2003, with a prevalence of 566 per 100–000 population (3), whereas a more recent Medicare claims analysis raised the annual prevalence to 701 per 100,000 population in subjects >65 years of age (4). Notably, the term bronchiectasis represents “an umbrella term” that in reality includes a variety of associated disorders (5), with several recent studies revealing significant worldwide etiologic diversity (6–8). Several attempts have been made to define clinical phenotypes in bronchiectasis (9, 10). However, taxonomy by cause appears to be clinically relevant only in a few conditions for which specific treatments are available (11).

Prior to our understanding of modern immunology, the most accepted model of bronchiectasis pathogenesis was the “vicious cycle” hypothesis proposed by Cole in 1986 (12). This model suggested that an initial insult in susceptible individuals may trigger chronic airway inflammation, leading to structural airway damage and abnormal mucociliary clearance. Abnormal inflammatory responses stimulated by subsequent infections may result in further airway damage and remodeling, perpetuating disease progression. More recently, the “vicious cycle” hypothesis has been replaced by the “vicious vortex” concept proposed by Flume et al. (13), which suggests a complex interaction of individual elements contributing to disease pathogenesis rather than a constant sequence of events.

The pathology of bronchiectasis may relate to a range of endogenous and exogenous insults, such as bacterial resistance against antibiotics, environmental pollutants, the presence and activity of comorbidities, and inflammatory endotypes, all of which contribute to the disease’s persistence and severity (14). Antibiotic overuse and/or misuse can lead to the emergence of resistant pathogens, that may persist within biofilms complicating treatment and maintaining chronic infection, inflammation and tissue damage (15). ​Environmental factors, particularly air pollution, also play a significant role in the progression of bronchiectasis. Exposure to pollutants such as particulate matter (PM_10_) and nitrogen dioxide (NO_2_), even at levels below established safety thresholds, can trigger systemic inflammation, exacerbating the disease and leading to adverse health outcomes and increased healthcare utilization (16). Multimorbidity in bronchiectasis is common and, although the specific impact on bronchiectasis pathogenesis is poorly. However, it is known that such comorbidities are associated with poorer clinical outcomes and increased mortality (17, 18). Understanding this multifactorial pathology will probably be essential for developing effective management and prevention strategies for bronchiectasis.

The inflammatory milieu in bronchiectasis is typically dominated by excessive accumulation of neutrophils, seemingly regardless of etiology (19–21). The progressive imbalance between pro- and anti-inflammatory cytokines and their inhibitors results in excessive accumulation of immune effector cells in the airways, leading to direct epithelial damage and ciliary dysfunction (22, 23). However, recent studies have indicated that airway eosinophilic inflammation is present in up to 20-30% of patients, predominating over or occurring concurrently with neutrophilic inflammation (24–26). Other immune cells, such as macrophages, are believed to play essential roles under certain circumstances (27), and significant numbers of peribronchial lymphoid aggregates containing B- and CD4+ T lymphocytes and germinal centers have been found in bronchial mucosal biopsies from patients with bronchiectasis (28–30). In contrast, patients with T-cell dysfunction are at increased risk of developing bronchiectasis (31, 32). **Table 1** summarizes the roles of key inflammatory cells in the pathogenesis of bronchiectasis.

**Table 1:** Inflammatory cells contributing to disease pathogenesis in bronchiectasis.

Cell Type	Key role	Major mechanisms/functions	Summary of findings in bronchiectasis (incl. key studies, methodology)
Neutrophils	First-line effector cells mediating microbial clearance and amplifying innate immune responses	Phagocytosis, degranulation of toxic mediators and proteases, NET formation, apoptosis	Normal or reduced oxidative burst; delayed and potentially aggravated pathogen clearance during exacerbations; increased protease release and NET formation; associated with tissue damage, airway remodeling, FEV₁ decline and exacerbations. Data from sputum, BALF, and blood samples (*in vivo*), neutrophil functional assays (*in vitro*) (33–42)
Eosinophils	Multipotent effector cells induced upon response to helminth parasite infections; type 2 immune response; contribution to epithelial dysfunction	Release of cytotoxic granules (MBP, ECP, EDN, EPX), production of chemokines, cytokines, growth factors	Elevated blood eosinophil counts and T2 inflammation in a subset of patients; linked to airway epithelial damage, mucus hypersecretion, and potential response to ICS and anti-IL5/ILRα therapies. Clinical cohort studies and sputum analysis (*in vivo*) (30, 43–54)
Th1 cells	Drivers of cell-mediated immunity and macrophage activation against intracellular pathogens, including bacteria, parasites, and viruses	Production of INF-γ, IL-2, TNF-β	Reduced Th1 responses in adults and children with severe bronchiectasis; shown in blood and tissue samples (*in vivo*) (55)
Th2 cells	Promoters of humoral immunity, to extracellular pathogens, including helminth parasites; orchestrators of T2 inflammation and allergy through B-cell activation and IgE class switching and eosinophilic recruitment and activation.	Production IL-4, IL-5, IL-10, IL-13	Increased T2 cytokines and specific antibodies against *P. aeruginosa, M. catarrhalis* and *S. maltophilia;* demonstrated in clinical studies (56–58)
Th17 cells	Regulators of mucosal immunity; inducers of neutrophilic recruitment and mucosal barrier reinforcement; defense against fungi, intracellular and extracellular bacteria; promotion of tissue inflammation and autoimmunity	Production of IL-17A, IL-17F, IL-21, IL-22, IL-26 and CCL20; upregulation of chemokines, MMPs	Elevated IL-17A and IL-23 in bronchiectasis compared to healthy and COPD controls; reduced Th17 inflammation with long-term macrolide therapy. Evidence from blood, BALF, and tissue samples (*in vivo*), cytokine analysis (*in vitro*) (59–65)
Tregs	Regulators of immune homeostasis through suppression of effector T-cell activity and inflammation; maintenance of mucosal homeostasis	Production of IL-10, TGF-β; inhibition of pro-inflammatory cytokines and chemokines	Decreased Treg numbers in bronchiectasis with coexisting COPD; relatively higher blood levels compared to CF patients. Finding from blood samples and flow cytometry (*in vivo*) (60–62)

Th, T helper; Tregs, T regulatory cells; NET, neutrophil extracellular trap; MBP, major basic protein; ECP, eosinophilic cationic protein; EDN, eosinophil-derived neurotoxin; EPX, eosinophil peroxidase; CCL, Chemokine Ligand; CLC/Gal-10, Charcot-Leyden crystal protein-galectin-10; IL, interleukin; IL-5Rα, Interleukin-5 Receptor-α; TNF, tumor necrosis factor; TGF, tumor growth factor; MIP-1,macrophage inflammatory protein-1; FEV1, forced expiratory volume in 1 second; T2, type 2; ICS, inhaled corticosteroids; BALF, bronchoalveolar lavage fluid; CF, cystic fibrosis; COPD, Chronic Obstructive Airway Disease.

There is increasing awareness of bronchiectasis as a clinical entity with new and developing treatment options. Therefore, expanding research into endotypes, which reflect the underlying pathophysiological mechanisms of the disease, may help us establish clinically adequate characterization of specific patient subpopulations. In this narrative review we provide an overview of immune cells and cytokine pathways involved in the pathogenesis of bronchiectasis not related to cystic fibrosis (CF). We also explore the role of neutrophilic inflammation as a key pathogenic feature associated with poor prognosis and the presence of an eosinophilic endotype in a significant subset of patients. The review also examines how bacterial infection can shift adaptive immunity from Th1 toward Th2 responses, the role of the airway microbiome-IL-17 axis in chronic inflammation and the potential protective role of Treg cells against excessive inflammation. Additionally, we explore the relationship between distinct inflammatory endotypes and microbiota alterations in severe bronchiectasis, as well as the potential benefits of targeting specific pathophysiological mechanisms in the management of bronchiectasis. We have performed searches in PubMed for bronchiectasis AND/OR inflammation AND/OR neutrophils AND/OR eosinophils AND/OR T helper lymphocytes AND/OR T regulatory cells between 1991 to present, with two-thirds of the articles published since 2016. Both eosinophilic and neutrophilic endotypes are covered, and data highlighting the potential protective and pathological roles of T-helper (Th) and T-regulatory (Treg) cells in this disorder are presented.

## 2 Inflammatory endotypes in bronchiectasis

### 2.1 The neutrophilic endotype

Neutrophils are the most abundant inflammatory cells in the airways of patients with bronchiectasis and are rapidly recruited from the bone marrow by several chemoattractants (such as IL-8/CXCL-8 and leukotriene B4) or proinflammatory cytokines (such as TNF-α) (66). In the lungs, neutrophils facilitate the rapid clearance of invading pathogens via a range of effector functions, including phagocytosis, degranulation of toxic mediators, and production of oxygen-free radicals (67, 68). Although phagocytosis is the primary means by which neutrophils contribute to bacterial killing, the formation of neutrophil extracellular traps (NETs) is also crucial for the trapping and elimination of pathogens through the release of extracellular neutrophil DNA webs containing large amounts of neutrophil serine proteases (SNPs) such as neutrophil elastase (NE), proteinase 3 (PR3), and cathepsin G (CatG), as well as antimicrobial peptides and platelets (69–72). Excessive levels of NETs in the airways promote an exaggerated inflammatory response, which is linked to disrupted mucociliary function, extracellular matrix degradation and hypersecretion of mucus with increased viscosity (33–35).

Neutrophilic inflammation and dysfunctional killing of pathogens are key elements in the pathogenesis of bronchiectasis (36). An early study reported that neutrophils preserve their normal phagocytic activity in the peripheral blood of patients with bronchiectasis (37). However, subsequent research has revealed that although phagocytosis does not differ between patients with bronchiectasis and healthy controls, neutrophilic oxidative burst may be reduced in the former (38). Indeed, this is compatible with the idea that the ability of airway neutrophils to kill bacteria is hampered and contributes to the pathogenesis in bronchiectasis. Prolonged neutrophil survival has been demonstrated in bronchiectasis, resulting in increased numbers of neutrophils, neither apoptotic nor necrotic, in the airways (39). Similarly, delayed apoptosis has been reported in patients with stable bronchiectasis, and this phenomenon may increase during exacerbations (40).

In bronchiectasis, airway neutrophilic inflammation, characterized by high concentrations of NETs, NE, and other neutrophil proteins, is associated with chronic airway infection, worse lung function, higher exacerbation rates, and more severe disease (20, 34, 41). Moreover, the antimicrobial peptide LL-37 may induce NET formation, and increased levels of this peptide have been associated with chronic *P. pseudomonas* infection, FEV₁ decline and higher exacerbation rates (42). Matrix metalloproteinases (MMP)-8 and -9 and pregnancy zone protein (PZP), a recently discovered NET-related protein, display increased levels in sputum from patients with bronchiectasis and have shown a positive correlation with clinical parameters, including radiological abnormalities, lung function, bacterial load, disease severity, and exacerbation risk (73, 74). Individuals with PI3K syndrome develop severe bronchiectasis, presumably because dysregulated PI3K activation contributes to immune dysfunction by augmenting neutrophil migration, degranulation, and ROS generation (20).

### 2.2 The eosinophilic endotype in bronchiectasis

Eosinophils are bone marrow-derived granulocytes that differentiate from CD34+ pluripotent progenitors and are regulated by several transcription factors, interleukins, and epithelium-derivate alarmins, including GATA-1, eotaxin-1, IL-5, IL-3, Granulocyte-Macrophage Colony-Stimulating Factor (GM-CSF), IL-33, IL-25, and thymic stromal lymphopoietin (TSLP) (75). Until recently, their role was restricted to host defense against parasitic infections (76). However, eosinophils are now recognized as key players in a range of airway disorders that can display pathological type 2 (T2) inflammation in local tissues, such as asthma, chronic rhinosinusitis with nasal polyps, eosinophilic granulomatosis with polyangiitis (EGPA) and a subset of patients with chronic obstructive pulmonary disease (COPD) (77). Degranulation of eosinophils, airway epithelium disruption, and increased mucus production may contribute to this association through the release of various mediators, such as chemokines, cytokines, and growth factors, which promote an intense airway inflammatory response (43). Moreover, their prolonged survival in inflamed tissues, mainly driven by IL-5, a cytokine produced by Th2 cells, mast cells, and type 2 innate lymphoid cells (ILC2s), is responsible for the persistence of inflammation, tissue damage, and airway remodeling in T2-related disorders (44, 45).

Recently, the eosinophilic endotype of bronchiectasis has been recognized, although the published data on these patients is limited. Along these lines, increased numbers of eosinophils have been found in the airway mucosa of patients with bronchiectasis compared with healthy controls (28). A small study has shown that eosinophilia, defined as ≥3% eosinophils in induced sputum, is associated with increased levels of T2 biomarkers, including FeNO and IL-13, although to a lesser degree compared to a severe refractory asthma reference population (25). Applying cutoff values of ≥3% and ≥300 cells/μL, a significant association between sputum and blood eosinophil counts has been demonstrated in approximately 20% of patients with bronchiectasis, despite the absence of classic eosinophil-driven comorbidities such as asthma and allergic pulmonary aspergillosis (ABPA) (24). These findings indicate that eosinophilic inflammation may play a significant role in a substantial proportion of bronchiectasis cases without coexisting asthma. This finding implies the potential for an alternative treatment strategy aligned with the current concept in COPD (46, 47). Recently, it was proposed that a T2-high endotype in bronchiectasis patients without concomitant asthma may be defined by FeNO levels of ≥25 ppb and a blood eosinophil count of ≥300 cells/μL (78). However, a recent study reported no significant differences in blood neutrophil levels between bronchiectasis patients with varying eosinophil levels (79). In addition, blood eosinophil counts of <100 cells/μL are linked to a more severe phenotype, probably reflecting a myeloid shift toward neutrophil production in response to persistent infection (80). Therefore, it remains unclear whether the presence of eosinophilia reflects predominant eosinophilic inflammation or a mixed inflammatory pattern owing to the dual nature of eosinophils (pro-inflammatory and anti-infective) (77).

Interestingly, there is evidence that certain airway bacteria promote eosinophilic inflammation in bronchiectasis. Recent studies have shown that specific airway pathogens, such as *S. aureus, Streptococcus* spp., and *P. aeruginosa*, are associated with higher blood eosinophil counts and shorter times to exacerbation (24, 81). In addition to reports that *P. aeruginosa* directly drives T2 inflammation in CF-related bronchiectasis (82), identification of this pathogen may explain the source of eosinophilia in the general bronchiectasis population, considering the known bactericidal capacity of eosinophils, thereby presenting a potentially treatable trait (48). There is also evidence that fungal exposure and sensitization may drive the T2 endotype in patients with bronchiectasis without evidence of ABPA, which can be identified by the presence of increased blood eosinophils, FeNO, and specific IgE levels (49).

## 3 T-helper and T-regulatory cells

### 3.1 T-helper and regulatory T (Treg) cells as immune response maestros

**Figure 1:**
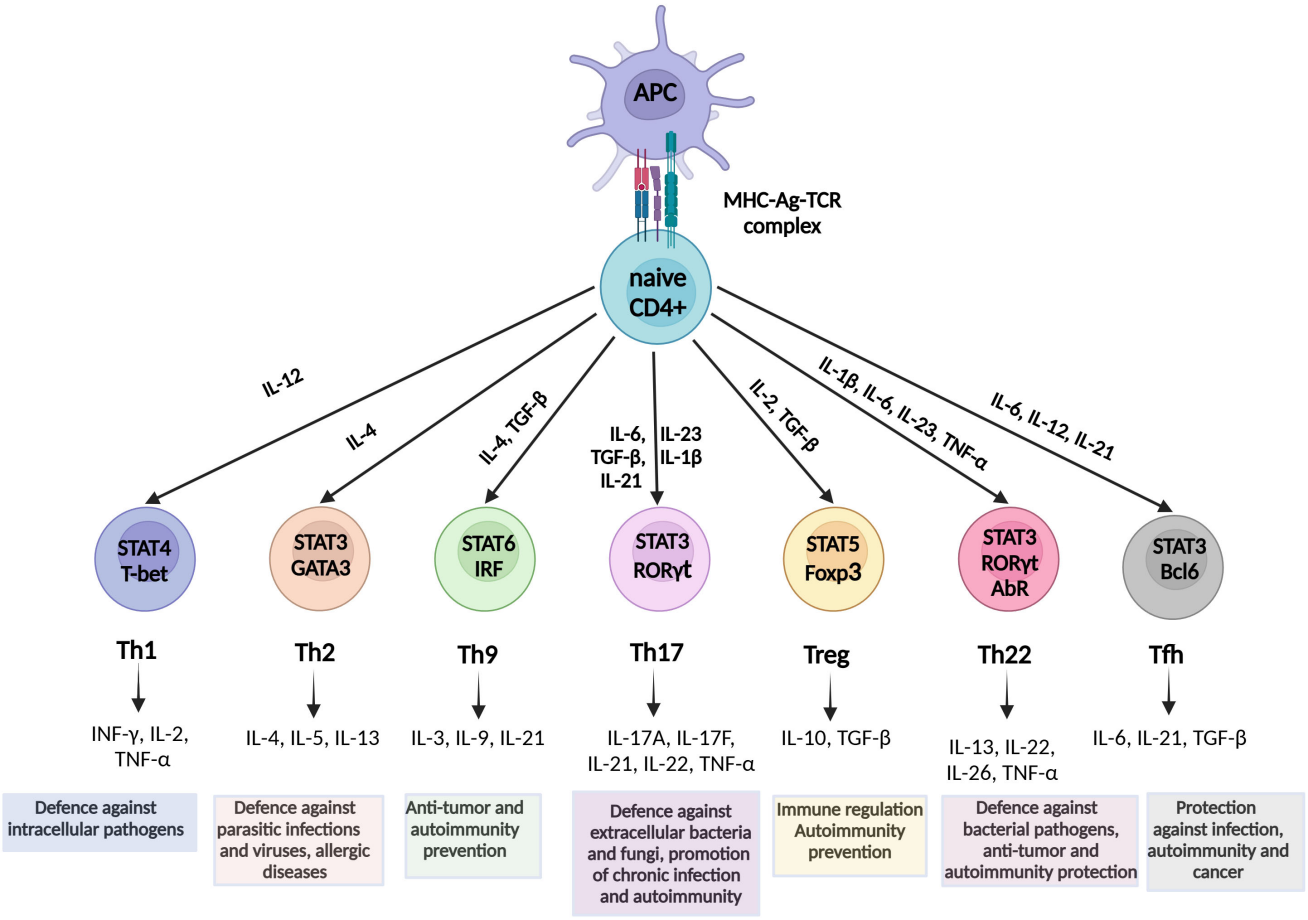
Schematic overview of Th cell differentiation from naive CD4+ T cells. Naïve CD4_+_ T cells differentiate into Th1, Th2, Th9, Th17, Th22, Treg, and Tfh subsets under the influence of specific cytokines and transcription factors. Each subset mediates distinct immune functions. Th1, intracellular pathogens and viruses defense; Th2, protection against parasitic infections, T2 immunity; Th9, anti-tumor activity, allergy; Th17, mucosal defense, chronic inflammation; Tregs, immune tolerance, autoimmunity prevention; Th22, barrier protection, autoimmunity prevention; and Tfh, B-cell activation and antibody production. Key signaling pathways and effector cytokines are indicated. STAT, Signal transducer and activator of transcription; INF, Interferon; IL, Interleukin; TNF, Tumor necrosis factor; TGF, Transforming growth factor; RORγt, related orphan receptor gamma; FOXp3, forkhead box P3; Tfh, T follicular helper.

T lymphocytes, commonly referred to as T cells, are critical players in the adaptive immune system, involved in the initiation of cell-based immune responses that protect the host against various diseases. These cells originate from thymocyte progenitors generated in the bone marrow and are categorized into four primary types: CD4+, CD8+, γδ T cells, and natural killer T (NKT) cells (50). Upon identification of cognate antigens by T cell receptors (TCRs) through MCH molecules on the surface of antigen-presenting cells (APCs), naïve CD4+ and CD8+ T cells undergo activation, clonal expansion, and differentiation, ultimately performing effector functions, including destruction of infected cells, cytokine production, and upregulation of immune responses (51). A subset of T cells transforms into memory T cells, which exhibit rapid effector responses upon re-encountering familiar antigens, thereby providing the host with robust and enduring protection (52). In parallel, naïve CD4+ T cells, also known as Th cells, have the potential to differentiate into functionally distinct subsets (Th1, Th2, Th9, Th17, Th22, follicular helper T (Tfh), and Treg) under the regulation of different transcription factors and signaling molecules, depending on the cytokine content of a certain microenvironment (53) (**Figure 1**).

After differentiation from naïve CD4+ T cells, Th1 cells provide phagocyte-dependent protective immunity against intracellular pathogens and viruses via IFN-γ, IL-2, and TNF-α production (54, 83). However, they also contribute to the resolution of acute tissue inflammation by inhibiting the powerful neutrophil chemoattractant IL-8 (84). In contrast, Th2 cells facilitate a phagocyte-independent protective mechanism associated with parasitic infections and allergic diseases via the production of IL-4, IL-5, IL-10, and IL-13 (85). Nonetheless, a reduced Th2 response reduces immune complex formation, thereby facilitating confinement of tissue damage (86). The Th9 CD4+ subgroup is believed to originate from Th2 cells, after their “reprogramming” by TGF-β and IL-4, and has been shown to promote antitumor protection and prevent autoimmunity (87). Notably, the proinflammatory Th22 subset participates in protective responses against bacterial pathogens and is associated with autoimmune pathogenesis and defense against tumors (88). Finally, Tfh cells play a critical role in protective immunity in response to various pathogenic infections, autoimmune disorders, and cancers (89, 90).

The current understanding is that Th17 cells play a vital role in preserving the integrity of mucosal epithelial barriers, while they are also crucial in protecting the host against bacterial and fungal mucosal infections through the secretion of Th17 cytokines (IL-17A, IL-17F, IL-21, IL-22, and IL-26), and potentially TNF-α and IL-6 upon certain stimulation (91, 92). These cells facilitate leukocyte tissue infiltration through upregulation of chemokines and metalloproteinases, however at the cost of promoting chronic inflammation and severe autoimmunity (93, 94).

The Treg subset is a discrete anti-inflammatory CD4+ subset that constitutes up to 10% of total CD4+ cells (95). An essential characteristic of Tregs is that they express FOXP3, a transcription factor critical for their development and function (96). According to their origin, FOXP3 Tregs can be divided into two major subsets: i) thymus-derived Tregs (tTregs), also known as natural Tregs, and ii) peripherally-induced Tregs (pTregs) (97, 98). These cells play a critical role in the maintenance of immune tolerance and have been shown to prevent the progression of several autoimmune and inflammatory diseases by suppressing numerous immune cells involved in innate and adaptive immune responses, such as B cells, CD4+ T cells, Th cells, CD8+ cells, NK cells, NKT cells, macrophages, dendritic cells, and neutrophils (99). Tregs perform their main functions through several mechanisms, such as cytokine secretion (mainly IL-10, but also TGF-β and IL-35), release of extracellular vesicles, granzyme/perforin mediated cellular cytolysis, cell-cell contact inducing modulation of dendritic cells, and metabolic perturbations (100). In addition, Tregs have been shown to suppress anticancer immune responses against autologous and tumor-expressing antigens by inhibiting the activation and differentiation of CD4+ helper and CD8+ cytotoxic T cells, thus contributing to tumor occurrence and progression (101, 102).

The balance between transcription factors Foxp3 and RORγt is critical in the Th17/Treg equilibrium, as Foxp3 activity may reduce Th17 cell differentiation through TGF-β-induced inhibition of RORγt function in the absence of other inflammatory stimuli (103). In contrast, IL-6, IL-21, and IL-23 promote Th17 versus Treg differentiation, favoring a proinflammatory environment (104).

### 3.2 The pathogenic potential role of T helper cells and the role of T regulatory cells

A growing body of evidence regarding the role of Th cells in CF is available, which may potentially provide valuable insights, even for patients with non-CF bronchiectasis. Preclinical studies have demonstrated that chronic infection with *P. aeruginosa* is characterized by a Th2-skewed response and further downregulation of the Th1 axis with decreased IFN-γ production, resulting in hampered ability to kill microbes (56, 57). In a murine model of CF, neutralization of endogenous IL-17 prior to infection with *P. aeruginosa* reduced bacterial load and neutrophil levels in the airway lumen (105). In contrast, transgenic mice lacking the IL-17RA receptor displayed a higher rate of infection and more significant mortality than wild-type mice after infection with two different *P. aeruginosa* strains, supporting that IL-17A plays a protective role in host defense against chronic pulmonary infection (106). However, higher peripheral blood Th17 signaling was strongly correlated with poorer lung function in a small study of adult CF patients compared to healthy controls (107). However, the role of Tregs in CF pathology remains to be elucidated. The results from one small study indicated a decreased peripheral blood Treg count in patients with CF colonized by *P. aeruginosa*. However, this decrease was not detected in subjects with intermittent *P. aeruginosa* infections, suggesting that chronic infection may be responsible for the Treg incapacity in CF (108).

In contrast to the case of Tregs, evidence regarding the role of specific Th cells in patients with non-CF-related bronchiectasis is scarce. A deficient Th1 response has been observed in adults and children with severe bronchiectasis (109). In contrast, a substantial Th2 response, resulting in increased levels of antibodies against *P. aeruginosa, M. catarrhalis*, and *S. maltophilia* has been reported in a controlled study on adult patients (38, 110). Notably, emerging evidence confirms the activation of T17 signaling in patients with bronchiectasis. In a small study on patients with stable bronchiectasis, investigators found significantly higher levels of several T17 cytokines in BALF than in healthy controls, including IL-17A and IL-23 (111). However, this study showed no association with clinical parameters or airway microbiology. Interestingly, while the gene expression of IL-17A in endobronchial biopsies was similar between the two groups, IL-1β levels were markedly higher in patients infected with *P. aeruginosa* and *H. influenzae*, suggesting a lesser involvement of Th17 in luminal host defense compared to innate neutrophilic activation. In contrast, higher levels of IL-17, IL-6 and Th17 cells, and lower levels of IL-10, TGF-β and Tregs in the peripheral blood were found in COPD patients with coexisting bronchiectasis and infection with *P. aeruginosa*, compared to patients with COPD only (112). Thus, Th17 cells may play a more prominent role in infection. Similarly, a recent study (113) demonstrated that patients with bronchiectasis unrelated to CF tend to have higher levels of Tregs in peripheral blood than CF patients, suggesting that chronic infection may impair Tregs in CF but not in non-CF bronchiectasis. Notably, treatment with a low-dose macrolide antibiotic (clarithromycin) significantly reduced both systemic and local Th17 responses in a small study of patients with bronchiectasis (114), suggesting that regulation of T17 signaling may be a beneficial, targeted treatment in these patients.

## 4 Interplay of inflammatory endotypes with microbiome in airway diseases

Bronchiectasis is characterized by alterations of bacterial airway microbiota, usually addressed as “dysbiosis”, including a reduced microbial diversity and increased quantities of opportunistic pathogens such as *P. aeruginosa, H. influenzae, and S. pneumoniae* (58). These pathogens perpetuate chronic inflammation through persistent colonization, biofilm formation, and immune evasion, thereby driving the vicious cycle of inflammation, tissue damage, and impaired mucociliary clearance (115). Thus, bacterial airway microbiota in bronchiectasis is defined by complex ecological dynamics, featuring dysbiosis and pathogen dominance, which significantly contribute to disease pathogenesis (116). Emerging evidence also underscores the potential roles of microbiota in terms of viruses and fungi as well, that may also impact bacteria and cytokine signaling, adding layers of complexity in bronchiectasis (117). Therapeutic strategies aimed at modulation of microbiota, such as inhaled antibiotics or probiotics, highlight the translational relevance of these interactions (118).

Microbial dysbiosis is a plausible factor in disease progression in patients with bronchiectasis, with genus diversity negatively correlating with disease severity, exacerbation frequency, and clinical outcomes, suggesting a bidirectional relationship between microbial community structure and host immune responses (55, 58). Bronchiectasis is characterized by frequent and diverse microbial infections and microbiota even in the stable state of the disease (59). The inflammatory response to dysbiosis may involve a complex network of cytokines that activate and recruit cells implicated in innate and adaptive immunity (60, 61). For instance, *P. aeruginosa* dominance is associated with increased sputum interleukin-1β, contributing to the airway injury (26). Early data from CF have indicated that dysregulated host-microbe interactions disrupt airway epithelial barrier integrity and promote a pro-inflammatory milieu, amplifying lung remodeling (62). Recent studies have highlighted the prognostic value of multi-omics data to reveal metabolic host-microbe disruptions, particularly in relation to microbial community dynamics during exacerbations (63, 64). Collectively, these studies emphasize the microbiome’s central role in bronchiectasis pathogenesis, offering insights for precision medicine approaches.

In patients with COPD who suffer from concomitant bronchiectasis, five endotypes have been described based on proteomic and microbiome profiling (65). Notably, these endotypes display distinct inflammatory, mucin, and microbiological characteristics. The first endotype was characterized by high microbial diversity and increased MUC5B, protease inhibitors, and immunoglobulin levels. The second featured *Haemophilus* microbiome predominance, elevated MMP8 and MMP9 levels, and a low MUC5AC/MUC5B ratio. The “infected-epithelial response” endotype, dominant by *Stenotrophomonas* and other *Proteobacteria*, was associated with increased concentrations of antimicrobial molecules, a high MUC5AC/MUC5B ratio, and low microbial diversity. The “dominant-NET” endotype was characterized by high NET proteins and *Proteobacteria* abundance, while the “Th2-driven” endotype featured T2 inflammation and increased dysbiosis. Similarly, in asthma-related bronchiectasis, at least two endotypes have been recognized and categorized into T2 high and T2 low subgroups, with further categorization based on specific T2 cytokines and biomarkers, allergy, and chronic bacterial infection (119).

A recent study investigated the interplay between the archetypes of inflammation signifying the key endotypes and microbiome characteristics in non-CF bronchiectasis patients from three European centers (26). Using cluster analysis of 33 sputum and serum inflammatory markers and 16S rRNA sequencing for microbiome analysis, the authors identified four distinct inflammatory endotypes associated with specific microbiome profiles. The more severe inflammatory clusters showed lower microbiome diversity and enrichment with Proteobacteria and *Pseudomonas*. Importantly, endotypes with severe neutrophilic and neutrophilic mixed with T2 inflammation were predictive of future exacerbation risk, suggesting their potential clinical utility in guiding targeted treatments.

Growing evidence suggests that sensitization to fungi may result in clinically relevant bronchiectasis with different endotypes. Allergic bronchopulmonary aspergillosis (ABPA) is a hypersensitivity response to *Aspergillus* spp., characterized by elevated blood IgE levels, peripheral eosinophilia and positive specific immunological tests for *Aspergillus*. The recent Cohort of Matched Asian and European Bronchiectasis (CAMEB) study identified two distinct sensitized immuno-allergic profiles in patients with bronchiectasis [68]. Patients with fungal-driven sensitization were characterized by specific responses to rAsp allergens and an airway rich in proinflammatory cytokines IL-1α, IL-1β, and TNF-α. In contrast, house dust mite sensitized patients showed increased levels of T2-related cytokines and airway eosinophilia, suggesting a potentially distinct treatable trait.

## 5 Discussion

An improved comprehension of the pathophysiology of bronchiectasis requires the specific understanding of three key components, impaired mucociliary function, inflammation, and infection, which together drive disease progression and clinical outcomes (13). Recent advances have significantly expanded our comprehension of bronchiectasis, moving beyond the traditional neutrophilic and eosinophilic dichotomy. These include severe neutrophilic, neutrophilic with T2 inflammation, Th2-driven, dominant-NET, infected-epithelial response, and a ‘shifted’ or mixed endotype exhibiting overlapping inflammatory features. In particular, research by Choi et al. (26) identified four inflammatory molecular endotypes in non-CF bronchiectasis, including severe neutrophilic, neutrophilic with T2 inflammation, and milder inflammatory clusters. Each of these endotypes is associated with distinct profiles of microbiota, as a result of dysbiosis, and relate to the risk of future exacerbations. This advanced endotyping approach, integrating immune profiles with microbiome features, marks a significant progression in disease understanding, with direct implications for risk assessment and personalized treatment strategies. Furthermore, the fungal sensitization endotypes reported by Mac Aogáin et al. (49), emphasize the significance of hypersensitivity responses as treatable traits, which may require specific antifungal or immunomodulatory treatments.

The role of the Th17/Treg axis may benefit from more investigation. Although Th17-driven inflammation is increasingly recognized for enhancing neutrophilic responses and contributing to tissue damage, the regulatory functions of Tregs and the consequences of Th17/Treg imbalance remain poorly defined in bronchiectasis. We propose that this type of dysregulation of immune response represents a novel mechanistic link to airway damage and disease progression, that warrants further investigation. Moreover, the emerging field of multi-omics research, which integrates transcriptomic, proteomic, metabolomic, and microbiome data, offers unique opportunities to define bronchiectasis endotypes more precisely and to identify possible therapeutic targets. By incorporating these perspectives, our work highlights important novel directions for advancing endotype-guided and precision medicine strategies in bronchiectasis.

As pointed out above, a central pathogenic mechanism in bronchiectasis is neutrophilic inflammation combined with dysfunctional bacterial killing, which perpetuates chronic airway damage and remodeling and is associated with worse outcomes (120). Neutrophil dysfunction, with reduced oxidative burst, increased viability, and delayed apoptosis of local neutrophils, has been demonstrated in bronchiectasis, providing a rationale for prolonged airway neutrophilia and impaired bacterial killing, thus contributing to further inflammation (121). Local accumulation of neutrophils in response to microbial dysbiosis and neutrophil dysfunction is common in several other chronic airway disorders, including COPD, CF, and a subgroup of patients with non-allergic asthma, thereby resembling bronchiectasis (122, 123).

Intriguingly, evidence from patients with CF suggests that neutrophils display a pro-survival cystic fibrosis transmembrane regulator (CFTR)-dependent phenotype, which prolongs their presence in the airways and induces NETs production (124). Similarly, non-CFTR-dependent inflammatory signals, including pro-inflammatory cytokines and lipopolysaccharides, have been associated with prolonged neutrophil survival in CF patients (125). Such changes in neutrophils’ metabolic pathways, including efferocytosis failure, primary or secondary neutrophil necrosis or some level of “reprogramming” of circulating blood neutrophils impairing their functional ability, have also been demonstrated in non-CF bronchiectasis patients, regardless of disease severity (40). However, whether neutrophil dysfunction in bronchiectasis results from an intrinsic defect or is provoked by a particular inflammatory milieu remains unclear.

An emerging insight in bronchiectasis pathogenesis is the recognition of an eosinophilic endotype, though its characterization remains under investigation.Existing evidence supports the existence of a distinct eosinophilic endotype of bronchiectasis, characterized by higher levels of FeNO and T2 cytokines in the airways, which is associated with poor clinical outcomes. However, a specific eosinophilic cutoff distinguishing a pathological state has not yet been determined, as other comorbidities characterized by increased T2 inflammation, such as asthma, chronic rhinosinusitis, ABPA, and eosinophilic COPD often coexist with bronchiectasis (7, 126–129). Although differentiating these diseases can be challenging, eosinophilic bronchiectasis is typically characterized by the absence of airway hyperresponsiveness (130), more severe dyspnea and more impaired respiratory function (78), and higher severity scores on various bronchiectasis indices (131). Nevertheless, the correlation between sputum and blood eosinophilia in bronchiectasis has been found to be weak (24), and stability of blood eosinophil numbers over time remains unclear (132). Eosinophil-specific inflammatory biomarkers, such as sputum eosinophil peroxidase, which significantly increase in severe bronchiectasis and during exacerbations requiring hospitalization, may better characterize this disease endotype (133). The role of Th cells in the dysregulated inflammatory environment that signifies bronchiectasis is yet to be fully understood. Persistent bacterial infection by certain pathogens has been shown to skew adaptive immunity from Th1 toward Th2 response, in an attempt to effectively control infection with respect to antibodies, innate cytokines, and chemokines production, while at the same time preventing further expansion of neutrophilic inflammation (110). Previous studies have shown that the airway microbiome-IL-17 axis is a critical regulator of chronic inflammation in bronchiectasis (29, 134). Th17 immune responses are known to activate neutrophils, playing an important role in host defense against bacteria while simultaneously contributing to persistent airway inflammation and bronchiectasis pathogenesis (115). However, the role of specific members of the airway microbiota in the modulation of IL-17/IL-23-type immunity in bronchiectasis remains unclear (135).

**Figure 2:**
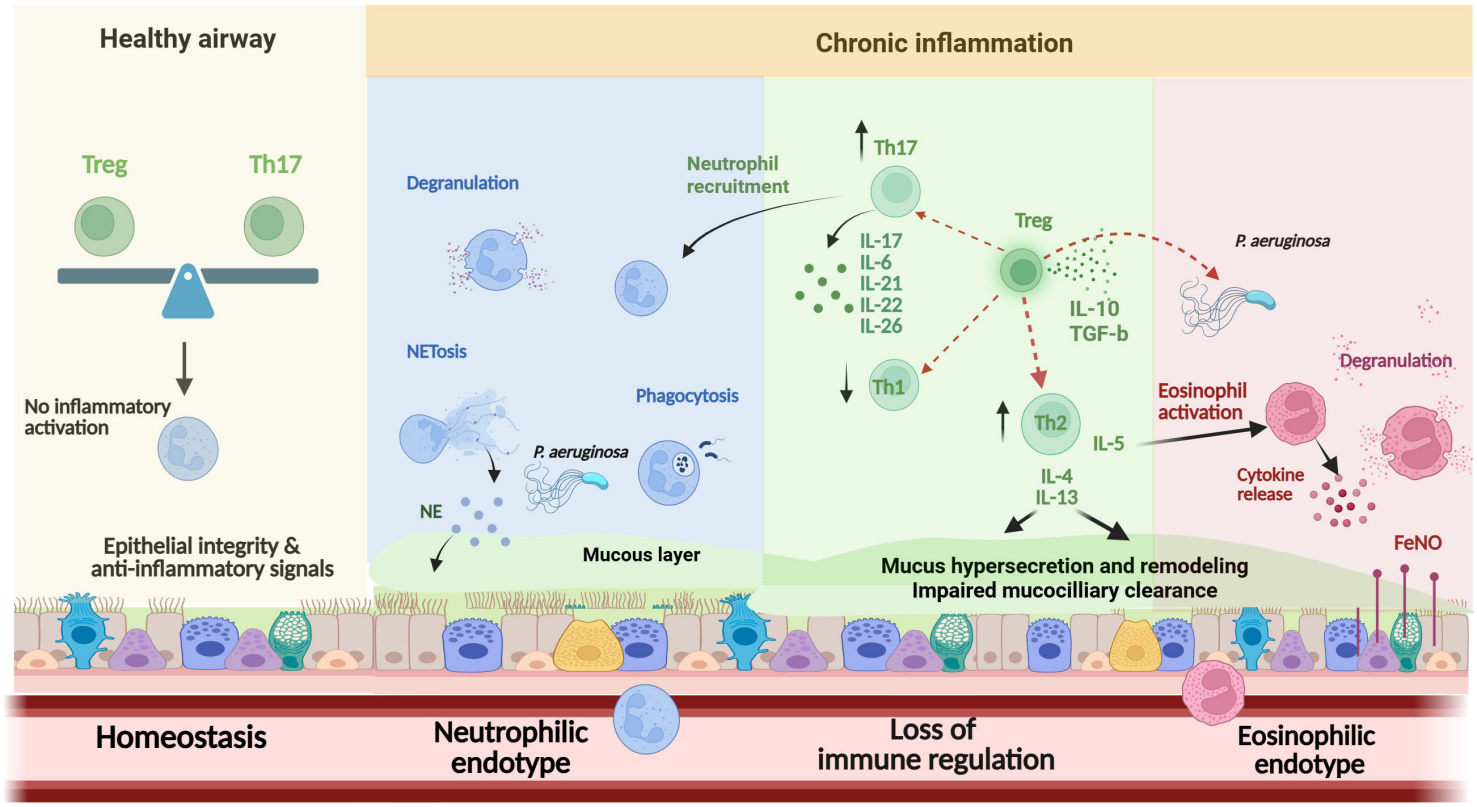
Overview of the main effector cells in the pathogenesis of bronchiectasis. Immune balance in healthy airways, maintained by Treg–Th17 homeostasis and epithelial-derived anti-inflammatory signals, prevents immune activation and preserves tissue integrity. In chronic dysbiosis, neutrophil dysfunction (including impaired oxidative burst, NETosis, and delayed apoptosis) and imbalances in Th1, Th2, and Th17 responses promote sustained inflammation. A distinct eosinophilic endotype, associated with T2 cytokines, elevated FeNO, and *P. aeruginosa* colonization, contributes to progressive epithelial damage and airway remodeling. The role of Tregs and the impact of Th17/Treg imbalance remain incompletely understood. Th, T-helper; Treg, T-regulatory; NE, Neutrophil elastase; NETs, neutrophil extracellular traps; IL, interleukin; FeNO, Fractional exhaled nitric oxide; T2, type 2 ↑, Increase; ↓, Decrease.

Clearly, a key knowledge gap is the role of Tregs in bronchiectasis and the balance between Th17 and Treg responses, which may hold the key to understanding immune dysregulation and tissue damage. In COPD, Tregs are reduced in number and dysfunctional, both in stable state and during exacerbations (136). However, further studies are required to confirm this concept in bronchiectasis. Since Tregs and Th17 cells display counterbalancing roles, with Th17 cells promoting inflammation and pathology and Tregs maintaining self-tolerance, Th17/Treg imbalance in bronchiectasis may result in significant immune dysfunction, permitting lung tissue damage and clinical deterioration (**Figure 2**).

Certain risk factors of the host may also affect the response of T-cell and myeloid cells involved in the pathogenesis of bronchiectasis. In inflammatory bowel disease, the lung-gut axis determines a shared immune system dysfunction associated with chronic lymphocytic inflammation (137). Individuals with PCD face genetically acquired impaired mucociliary clearance, which increases their susceptibility to infections and drives local neutrophil accumulation (138). Patients with immunodeficiencies may experience persistent infections due to impaired local protective humoral immunity caused by abnormalities in B- and T-cell functions (139–141). Likewise, in Job’s syndrome, a disorder characterized by bronchiectasis, persistent lung infections, and increased IgE levels, a STAT3 gene mutation impairs IL-17 production, affecting the production of a diverse array of cytokines (142).

While most research to date has focused on CF, non-CF bronchiectasis, despite being more heterogeneous and increasingly acknowledged, has not been equally explored. The limited available data suggest that chronic infection, airway colonization, and persistent inflammation may influence T-cell responses in non-CF bronchiectasis. However, these conclusions are often drawn from small cohorts or animal models, urging the need for well-designed, disease-specific studies to understand how T-helper and Treg cell dysregulation contributes to disease progression and treatment response in this population.

The primary goal of current bronchiectasis management is to address the underlying cause of the disorder by enhancing mucociliary clearance, managing infections, and effectively preventing and treating associated complications (143). However, the emerging identification of several endotypes in bronchiectasis, especially those involving specific immune cells and their cytokine signaling, has highlighted the need for more targeted approaches that can inform treatment decisions and improve clinical outcomes in specific patient subpopulations (144). Furthermore, a recent study identified distinct inflammatory molecular endotypes in bronchiectasis patients, demonstrating that these endotypes are associated with different microbiome profiles and future exacerbation risks, emphasizing that recognizing of various inflammatory processes at an individual patient level is crucial for developing management plans that prevent exacerbations and tailor treatments effectively (26). Inflammatory mechanisms, such as neutrophil extracellular traps and eosinophilia, are instrumental in defining these endotypes, and biomarkers related to these processes may be useful in guiding therapies and enhancing the success of randomized trials (126).

Targeting neutrophilic inflammation in bronchiectasis has consistently posed a challenge as many approaches have aimed to prevent neutrophil recruitment to the lungs, with evident risks in a chronic disorder where bacteria operate. CXCR2 antagonists have effectively achieved this outcome but have been associated with increased infections and exacerbations (145). In contrast, targeting metabolic reprogramming, such as with 5’ adenosine monophosphate-activated protein kinase (AMPK) activators, which modulate multiple intracellular metabolic pathways, including glycolysis, and reverse phagocytic dysfunction and NET formation, could potentially reverse neutrophil dysfunction and dysregulated inflammation (36). Brensocatib, a reversible inhibitor of CatC that blocks the activation of NSPs in the bone marrow during neutrophil maturation, has shown broad anti-inflammatory effects beyond its known impact on serine proteases, resulting in significant improvements in clinical outcomes, particularly reductions in rate of pulmonary exacerbations and FEV_1_ decline (146–149). In two recent phase II randomized, placebo-controlled, dose-finding studies, the novel selective inhibitors of CatC, BI 1291583 and HSK31858, were found to be well-tolerated and effective in reducing the risk of exacerbation in adults with bronchiectasis (150, 151). Focusing on T2 inflammation in individuals with bronchiectasis offers promising potential for enhancing clinical outcomes, aligning with the move toward precision medicine in managing bronchiectasis (152). Although recent guidelines do not recommend the routine administration of ICS in patients with bronchiectasis, emerging evidence suggests that their use may be associated with a reduced frequency of exacerbations in patients with elevated blood eosinophil levels (153). Of particular significance is the potential for eosinophils and FeNO to serve as biomarkers of IL-5, IL-4Ra, or TSLP-driven inflammation, as these cytokines may constitute targets for future biological therapies, regardless of the co-existence of asthma or COPD. So far, the effectiveness of anti-IL-5 or anti-IL-5 receptor monoclonal antibodies in severe eosinophilic bronchiectasis has been documented in a case series, which demonstrated marked improvements in exacerbation rates and lung function after six months of treatment (154). A phase III, randomized, placebo-controlled trial (NCT05006573) was conducted to evaluate the efficacy and safety of benralizumab in adults with bronchiectasis and eosinophilic inflammation. However, due to its early termination, there is no available published data or outcomes, highlighting the need for further research in this area (155).

Furthermore, recent evidence from CF has demonstrated that CFTR-targeted medications can reduce eosinophils and mucus plugs and reverse exaggerated airway enlargement, suggesting a potential novel therapeutic approach for patients with non-CF bronchiectasis with evidence of underlying CFTR dysfunction (156).

Finally, elucidating the contribution of the Th17/Treg axis in the pathogenesis of bronchiectasis may reveal novel disease-modulatory therapies. In this context, it is interesting that macrolides, which are part of validated and standardized therapy, do reduce both neutrophilic and Th17-driven inflammation (20, 143). In fact, there is evidence that anti-IL-17 treatment reduces LPS-induced inflammation in murine asthma models (157, 158) and in an elastase-induced murine emphysema model (159). Future research on targeting Th17/Treg effector cells and related cytokines and therapeutic strategies that focus on restoring the Th17/Treg axis balance may serve as promising future targets for modulating lung inflammation and preventing or delaying tissue damage in bronchiectasis (160). This is especially true considering the increasing interest and potential therapeutic role of Tregs in autoimmune and inflammatory diseases, including cancer (161). Taken together, these insights highlight the complexity of bronchiectasis pathogenesis and the urgent need for endotype-guided therapeutic strategies.

## 6 Conclusions

Patients with bronchiectasis exhibit several endotypes with specific inflammatory characteristics, reflecting the complex interplay between dysfunctional host immunity, pathogens, and environmental factors. Identified endotypes include not only the classic neutrophilic and eosinophilic types, but also mixed or ‘shifted’ endotypes, Th2-driven and dominant-NET profiles, each with distinct immunopathological features. The key conclusions of this review are summarized below:

- Precise determination of endotype is critical for characterizing and subtyping bronchiectasis, given that certain inflammatory profiles are strongly associated with clinical phenotypes, disease severity, and clinical outcomes.
- Recognition of inflammatory endotypes provides a promising tool for developing targeted therapies, necessitating the integration of basic mechanistic investigations, translational studies, and clinical validation of candidate biomarkers.
- Recent advances in multi-omics and immunophenotyping offer unique prospects to define bronchiectasis endotypes with greater precision and to identify feasible therapeutic targets.
- The role of T-helper and T-regulatory cells remains underexplored in non-CF bronchiectasis; addressing these gaps through well-designed, disease-specific studies is essential for developing tailored immunomodulatory treatments.
